# Health-related Quality of Life (HRQOL) Decreases Independently of Chronic Conditions and Geriatric Syndromes in Older Adults With Diabetes: The Fujiwara-kyo Study

**DOI:** 10.2188/jea.JE20130131

**Published:** 2014-07-05

**Authors:** Satoko Nezu, Nozomi Okamoto, Masayuki Morikawa, Keigo Saeki, Kenji Obayashi, Kimiko Tomioka, Masayo Komatsu, Junko Iwamoto, Norio Kurumatani

**Affiliations:** 1 Department of Community Health and Epidemiology, Nara Medical University School of Medicine, Kashihara, Nara, Japan; 1 奈良県立医科大学地域健康医学教室; 2 Sakai City Mental Health Center, Sakai, Osaka, Japan; 2 堺市こころの健康センター; 3 Faculty of Nursing, Nara Medical University School of Medicine, Kashihara, Nara, Japan; 3 奈良県立医科大学看護学科; 4 Department of Nursing, Tenri Health Care University, Tenri, Nara, Japan; 4 天理医療大学看護学科

**Keywords:** diabetes, older adults, geriatric syndromes, chronic conditions, SF-36

## Abstract

**Background:**

Very few studies have investigated the association between diabetes and impaired health-related quality of life (HRQOL) in older adults, independent of chronic conditions and geriatric syndromes.

**Methods:**

We conducted a self-administered questionnaire survey and structured interviews with 3946 people aged 65 years or older to obtain medical histories of diabetes, chronic conditions, and geriatric syndromes. Blood tests were performed to measure glycated hemoglobin (HbA1c) and plasma glucose levels. HRQOL was evaluated using the Medical Outcomes Study 36-Item Short-Form General Health Survey (SF-36), and multiple logistic regression analysis was used to calculate adjusted odds ratios and 95% CIs for low HRQOL.

**Results:**

A total of 3521 participants had not received a physician diagnosis of diabetes. Of these, 2345 participants with an HbA1c less than 5.7% were defined as the referent group. As compared with the referent group, 1029 participants with an HbA1c of at least 5.7% but less than 6.5% showed no significant decrease in QOL on the SF-36 physical, mental, and role component summaries, after adjustment for chronic conditions, geriatric syndromes, and other potential confounders. However, 572 patients who had received a physician diagnosis of diabetes and/or had an HbA1c of 6.5% or higher had a significantly higher adjusted odds ratio (1.48; 95% CI, 1.18–1.84) for the low physical component summary. No significant differences in relation to glycemic control, treatment regimen, or diabetes duration were found in any of the 3 component summaries among the 425 participants who were undergoing diabetes treatment.

**Conclusions:**

Older Japanese adults with diabetes had decreased physical QOL, independent of chronic conditions and geriatric syndromes.

## Abstract (ja)

**背景：:**

高齢者の糖尿病が慢性疾患や老年症候群と独立して健康関連QOL（HRQOL）低下に関連しているか否かを検討した研究は極めて乏しい。

**方法：:**

65歳以上の高齢者3,946人を対象に、自記式質問紙と面接調査で糖尿病と慢性疾患および老年症候群に関する病歴を得るとともに、HbA1cと血糖値の血液検査を実施した。HRQOLはSF-36（包括的健康関連QOL評価尺度）を用いて評価し、低HRQOLに関する調整オッズ比と95％信頼区間を多重ロジスティック回帰分析で算出した。

**結果：:**

全対象者のうち3,521人は医師から糖尿病と診断されたことはなく、そのうちのHbA1c<5.7%であった2,345人を基準群とした。HbA1c ≥5.7% かつHbA1c <6.5% であった1,029人の基準群に対する低HRQOLの調整オッズ比を慢性疾患、老年症候群やその他の交絡要因を調整して求めたところ、SF-36の3種類のcomponent summaryであるPCS（身体的側面の健康観）、MCS（精神的側面の健康観）、RCS（役割/社会的側面の健康観）のいずれも有意な上昇はみられなかった。しかし、医師に糖尿病と診断されているかまたはHbA1c ≥6.5%であった572人の糖尿病群では、PCSに関する調整オッズ比は 1.48 (95% CI 1.18-1.84)と有意に高値であった。一方、糖尿病と診断され治療をしていた425人では、血糖コントロール、治療方法、罹病期間の違いによりPCS、MCS、RCSで低QOLに関する有意なオッズ比の上昇はみられなかった。

**結論：:**

糖尿病罹患高齢者の身体的QOLは慢性疾患や老年症候群と独立して低下していた。

## INTRODUCTION

In conjunction with the increase in the global elderly population, diabetes among older adults is a growing health burden worldwide.^1^ Japan has one of the oldest populations in the world, and 16.2% of community-dwelling individuals aged 70 years or older (22.6% of men and 11.6% of women) receive treatment for diabetes and/or have an HbA1c of 6.5% or higher.^2^ Some guidelines and reviews^3^^–^^5^ have emphasized the importance of efforts to maximize health-related quality of life (HRQOL) in geriatric diabetes care, because HRQOL is an outcome measure used to monitor diabetes burden and poor HRQOL is associated with adverse outcomes, including disease progression, hospitalization, and mortality.^6^^,^^7^ Accordingly, decreased HRQOL among older adults with diabetes is a major healthcare concern.

The QOL of people with diabetes is associated with (1) demographic characteristics such as age, sex, education, and ethnicity, (2) disease-specific attitudes including diabetes self-efficacy, locus of control, and social support, (3) and disease-specific medical factors, including type and duration of diabetes, treatment regimen, level of glycemic control, and presence of complications.^8^ In addition to these factors, potential confounders commonly associated with the complex health status of elderly individuals,^9^ such as chronic conditions and geriatric syndromes, should be considered in any investigation of the impact of diabetes on QOL in elderly populations.

Many studies have investigated the HRQOL of people with diabetes across a wide age range, but few have specifically targeted older adults. Some showed correlations between low HRQOL and comorbidities,^10^ diabetic complications,^11^^,^^12^ and hypoglycemia.^13^ Others compared HRQOL in community-dwelling elderly people with or without diabetes.^14^^,^^15^ These studies, however, did not fully account for specific confounding factors that influence HRQOL among older adults. Therefore, questions remain regarding whether diabetes alone contributes to decreases in HRQOL and the extent to which diabetes affects HRQOL.

To investigate the association between diabetes and impaired HRQOL among community-dwelling older adults, we conducted a large-scale cross-sectional dataset analysis in which chronic conditions and geriatric syndromes were considered as potential multidimensional confounders.

## METHODS

### Subjects

The subjects were independent community-dwelling older adults in Japan who participated in the Fujiwara-kyo study,^16^^,^^17^ a population-based cohort study targeting residents of 4 cities in Nara, Japan. Fujiwara-kyo is the name of the first ancient capital of Japan and the place where most participants lived. We aimed to establish a scientific basis for increasing healthy life-years and promoting QOL among the elderly. Potential participants were recruited with the cooperation of local resident associations and elder clubs. Eligible participants were aged 65 years or older, living in their own homes, and able to walk independently and provide written informed consent. We recruited a total of 4427 (2174 men and 2253 women) participants, all of whom underwent baseline examinations between June 2007 and October 2008.

The protocols of this study were approved by the Medical Ethics Committee of Nara Medical University. All participants provided written informed consent before study participation.

### Questionnaire

Each participant completed a self-administered questionnaire consisting of 250 items, including HRQOL, histories of diseases, geriatric syndromes, and demographic characteristics. Responses to the questionnaire were confirmed through interviews by trained research staff at the baseline examinations.

### Measurements

Venous blood samples were collected after overnight fasting. Glycated hemoglobin (HbA1c; National Glycohemoglobin Standardization Program values), plasma glucose, and serum creatinine were measured at a commercial laboratory (SRL Co. Inc., Tokyo, Japan) using a standard clinical chemistry analyzer. After the participant had rested for 5 minutes in a sitting position, an automatic sphygmomanometer was used to measure blood pressure twice, with a 1-minute interval between measurements. The average of the 2 readings was used in the analysis. Body mass index (BMI) was calculated as body weight (kg) divided by the height squared (m^2^). Weight and height were measured at the baseline examination.

### Definition of diabetes

Participants were asked whether they had received a diagnosis of diabetes from their physicians and, if they had, when the diagnosis was given and the details of the treatment regimen (ie, lifestyle modification, oral medication, and/or insulin therapy). Medications were confirmed by examining the prescription notes of physicians, which were provided by the individual participants. In this study, the diabetes group included participants with physician-diagnosed diabetes who were receiving medical treatment (diagnosed diabetes), and the undiagnosed diabetes group included those without a physician diagnosis of diabetes but with an HbA1c of 6.5% or higher at baseline.^18^ On the basis of HbA1c levels, the remaining participants were classified into 2 non-diabetes groups: the referent group (HbA1c <5.7%) and the high HbA1c group (HbA1c 5.7% to <6.5%). An HbA1c of 6.5% or higher is a criterion for a diabetes diagnosis, and an HbA1c of 5.7% to less than 6.5% is associated with an increased risk of diabetes.^18^ Poor glycemic control was defined as an HbA1c of 7.0% or higher.^3^^,^^4^

### HRQOL assessment

HRQOL was assessed using the Japanese version of the Medical Outcomes Study 36-Item Short-Form General Health Survey Version 2.0 (SF-36 v2), a generic non-disease–specific HRQOL questionnaire with established reliability and validity.^19^ The SF-36 consists of 36 items that aim to measure a person’s perspective on health status during the past 4 weeks. The items are scored in 1 of 8 SF-36 scales, namely, physical functioning (PF), role physical (RP), bodily pain (BP), general health (GH), vitality (VT), social functioning (SF), role emotional (RE), and mental health (MH) (range, 0 to 100). Each score for the 8 scales is then transformed to a mean of 50 and a standard deviation of 10, based on the 2007 Japanese population norms.^20^ The 2-component SF-36 model, which consists of a physical component summary (PCS) and a mental component summary (MCS), is derived from the 8 scales from Western populations, but this model cannot be applied to Asian populations, including the Japanese population.^21^ A recent study found that a 3-component model that adds role component summary (RCS) to PCS and MCS adequately measured HRQOL among Japanese.^22^ A factor analysis of the 8 scales indicated that (1) PCS is characterized by physical health condition, which is dominantly associated with PF, RP, BP, and GH, (2) MCS shows mental health condition, which is dominantly associated with VT, MH, SF, and GH, and (3) RCS implies role and social functioning, which is dominantly associated with RE, RP, and SF.

### Covariates

On the basis of the self-administered questionnaire survey and structured interviews, we confirmed past and present histories of 3 diseases: stroke with clinical symptoms, acute myocardial infarction (AMI), and cancer of any kind. We also assessed 6 chronic conditions: hypertension, low estimated glomerular filtration rate (eGFR), overweight (BMI ≥25 kg/m^2^), sleep disturbance, poor vision, and poor hearing. Hypertension was defined as use of antihypertensive medications or a systolic blood pressure of 140 mm Hg or higher and/or a diastolic blood pressure of 90 mm Hg or higher at baseline. eGFR was calculated with serum creatinine using a formula for Japanese populations; low eGFR was defined as a value less than 60 mL/min/1.73 m^2^.^23^ Sleep disturbance was evaluated using the Pittsburgh Sleep Quality Index (PSQI; score range 0–21), and those with a score of 5.5 or higher were considered to have a sleep disturbance.^24^ Vision and hearing were subjectively evaluated using 2 corresponding questions from the Japanese version of the 15D questionnaire,^25^ a self-administered 15-dimensional instrument for measuring HRQOL in adults.

Geriatric syndromes comprised 5 items: depressive symptoms, cognitive impairment, underweight, falls, and urinary incontinence.^4^^,^^5^ Depressive symptoms were evaluated using the 15-item short form of the Geriatric Depression Scale (GDS15; score range 0–15), and a score of 6 or higher was defined as presence of depressive symptoms.^26^ Cognitive function was evaluated using the Mini-Mental State Examination (MMSE; score range 0–30), and a score 23 or lower was considered to indicate the presence of cognitive impairment.^27^ Underweight was defined as a BMI of 18.5 kg/m^2^ or lower. Urinary incontinence was considered present when the participant experienced involuntary leakage of urine or a sudden urge to urinate that culminated in accidental leakage.^28^ Falls were defined as having more than 1 fall during the past year.

Psychosocial factors included education (≤12 or >12 years), living alone, and social support. Social support was evaluated by the Jichi Medical School Social Support Scale for the Japanese,^29^ a 28-item questionnaire used to measure the availability of social support by a spouse, family members, or friends. The cutoff point for each subscale was set as the mean value.

### Statistical analysis

The Mann-Whitney test was used to compare medians between diabetes and non-diabetes groups. The Mantel-Haenszel test was used to compare the prevalence of categorical variables adjusted for sex and 5-year age group based on the distribution of the non-diabetes group.

The impact of diabetes on HRQOL was evaluated by multiple logistic regression analysis using the forced entry method. The prevalence odds ratio (OR) was expressed as a point estimate with a 95% CI. Dependent variables were low PCS, MCS, and RCS, each defined by a score less than the 25th percentile score. Independent variables included past or present history of stroke, AMI, and cancer, chronic conditions (hypertension, low eGFR, overweight, sleep disturbance, poor vision, and poor hearing), geriatric syndromes (depressive symptoms, cognitive impairment, underweight, urinary incontinence, and falls), and psychosocial factors (living alone; social support by spouse, family members, or friends; and education). These variables were categorized as present/absent or by using their respective cutoff values. For each dependent variable, multiple logistic regression analysis was performed using only independent variables that were significantly associated with the corresponding dependent variable, after adjusting for sex and age (1-year units), at *P* < 0.1.

All statistical analyses were performed using SPSS for Windows 7 (Version 17). The null hypothesis was rejected when the 2-sided *P*-value was greater than 0.05.

## RESULTS

After excluding nonrespondents to the SF-36 (*n* = 91) and participants with missing data on blood testing (*n* = 22), geriatric syndromes (*n* = 232), or other items necessary for analysis (*n* = 136), data from 3946 (89.1%) participants were analyzed. All participants were Japanese, median age was 72.0 years (interquartile range, 68.0–76.0), and 50.4% were male. Of these, 572 participants were assigned to the diabetes group (425 with diagnosed diabetes, 147 with undiagnosed diabetes), and the remaining 3374 to the non-diabetes group (high HbA1c, 1029; referent, 2345), based on the definitions described in the Methods. Of the total, 48 individuals used insulin regularly.

As shown in Table 1, the proportion of men was larger and HbA1c levels and plasma glucose were higher in the diabetes group as compared with the non-diabetes group. In addition, the prevalences of stroke, AMI, overweight, depressive symptoms, urinary incontinence, and absence of spousal support were significantly higher in the diabetes group. In contrast, the prevalence of underweight was significantly higher in the non-diabetes group. The median PCS score was significantly lower in the diabetes group than in the non-diabetes group, but median MCS and median RCS scores did not significantly differ. Within the diabetes group, no significant differences were observed between participants with diagnosed diabetes and those with undiagnosed diabetes in any assessed parameter, including HbA1c (median: 6.7% vs 6.8%), with the exception of the prevalences of stroke (10.1% vs 4.1%), AMI (6.4% vs 3.4%), and low eGFR (30.8% vs 17.7%).

**Table 1.** Demographic characteristics of participants in diabetes and non-diabetes groups

		Diabetes group^a^ (*n* = 572)		Non-diabetes group (*n* = 3374)		*P*-value
Demographics						
Age		72.0	69.0–76.0	72.0	68.0–75.0	0.230
Sex	Male	366	64.0%	1621	48.0%	<0.001
Blood examination						
HbA1c	%	6.8	6.4–7.4	5.5	5.2–5.6	<0.001
Plasma glucose	mmol	6.8	5.8–8.7	5.2	4.9–5.6	<0.001
Past or present history						
Stroke	Yes	50	8.2%	186	5.5%	0.035
AMI	Yes	32	5.1%	71	2.1%	0.003
Cancer	Yes	68	11.2%	307	9.1%	0.148
Chronic conditions						
Hypertension^b^	Present	429	74.5%	2297	68.1%	0.098
Low eGFR	<60 ml/min/1.73 m^2^	157	26.1%	838	24.8%	0.342
Overweight	BMI ≥25 kg/m^2^	167	31.2%	731	21.7%	<0.001
Sleep disturbance	PSQI ≥5.5	205	37.1%	1160	34.4%	0.244
Poor vision	Present	14	2.6%	80	2.3%	0.374
Poor hearing	Present	51	8.3%	337	10.0%	0.152
Geriatric syndromes						
Depressive symptoms	GDS ≥6	108	20.0%	494	14.6%	0.013
Cognitive impairment	MMSE ≤23	39	6.2%	163	4.8%	0.165
Underweight	BMI <18.5 kg/m^2^	21	4.1%	228	6.8%	0.007
Urinary incontinence	Present	133	26.3%	689	20.4%	0.020
Falls	Present	123	22.9%	662	19.6%	0.128
Psychosocial factors						
Living alone	Present	45	9.7%	370	11.0%	0.275
Support by spouse	Absent	126	26.6%	789	23.4%	0.017
Support by family members	Absent	172	29.6%	859	25.5%	0.088
Support by friends	Absent	161	26.6%	817	24.2%	0.225
Education	≤12 years	408	71.9%	2422	71.8%	0.400
Scores for the SF-36 component summary						
PCS: Physical Component Summary		46.0	38.2–51.5	48.0	41.1–53.4	<0.001
MCS: Mental Component Summary		56.3	50.1–61.6	56.5	50.6–61.7	0.202
RCS: Role Component Summary		51.2	41.4–57.0	50.1	41.7–55.8	0.114

Data are expressed as number of corresponding participants (%) or median (25th–75th percentiles).

Percentages were adjusted for sex and 5-year age group, based on the distribution of the non-diabetes group, and tested using the Mantel-Haenszel method.

^a^Participants with a diabetes diagnosis and/or HbA1c ≥6.5%.

^b^Participants taking antihypertensive medications, or with systolic blood pressure ≥140 mm Hg or diastolic blood pressure ≥90 mm Hg.

AMI: acute myocardial infarction, eGFR: estimated glomerular filtration rate, BMI: body mass index, PSQI: Pittsburgh sleep quality index, GDS: geriatric depression scale, MMSE: mini-mental state examination.

The Figure shows the medians and 25th percentile scores for each of the 3 component summaries by sex and 5-year age group. PCS and RCS scores decreased with age, whereas MCS scores increased. In general, median and 25th percentile scores were higher in men than in women within the same age group, which suggests that sex and age adjustments are necessary when comparing the 3 component summaries between sexes and across different age-based populations.

**Figure.**
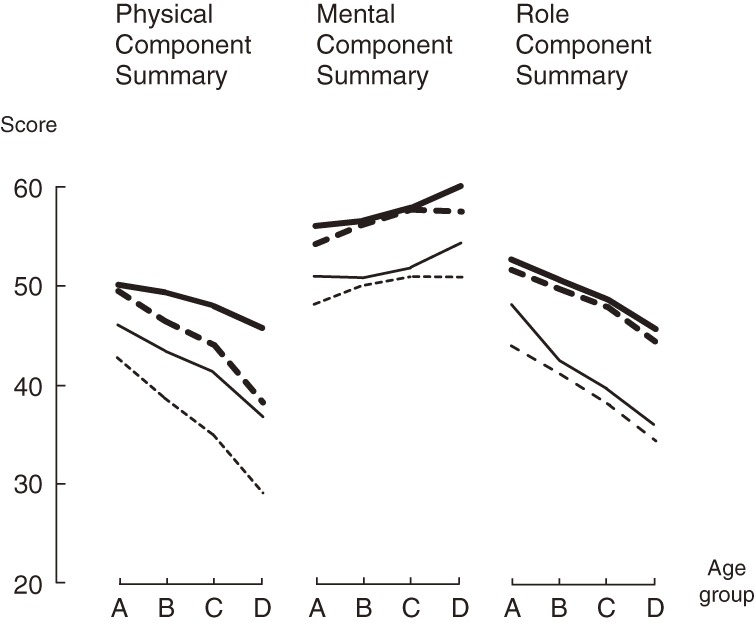
Median and 25th percentile scores for the 3 SF-36 component summaries, by sex and 5-year age group. Thick lines show medians in men; dotted thick lines, medians in women; thin lines, 25th percentile scores in men; and thin dotted lines, 25th percentile scores in women. Age groups: A, 65 to <70; B, 70 to <74; C, 75 to <80; and D, ≥80.

Table 2 shows adjusted ORs of independent variables for low PCS, MCS, and RCS. The ORs for low PCS, MCS, and RCS were not significantly higher in the high HbA1c group than in the referent group. However, in the diabetes group, the age- and sex-adjusted OR (1.73; 95% CI 1.40–2.14) for low PCS, but not for low MCS or low RCS, was significantly higher than unity. The OR remained significant (1.48; 95% CI 1.18–1.84) even after adjusting for all possible confounders. Additionally, the ORs for low PCS, MCS, and RCS were higher for some diseases, chronic conditions, geriatric syndromes, and psychosocial factors.

**Table 2.** ORs and 95% CIs for low PCS, MCS, and RCS, mutually adjusted for independent variables without an asterisk in each component summary

Independent variables		Physical component summary						Mental component summary						Role component summary					
		
		OR	95% CI		OR	95% CI		OR	95% CI		OR	95% CI		OR	95% CI		OR	95% CI	
Diabetes status	Referent (2345)	1			1			1			1			1			1		
	High HbA1c (1029)	1.18	0.99	1.40	1.11	0.93	1.33	0.94	0.79	1.12	0.95	0.79	1.14	0.92	0.77	1.09	0.93	0.77	1.11
	Diabetes (572)	1.73	1.40	2.14	1.48	1.18	1.84	1.17	0.95	1.44	1.03	0.82	1.30	1.01	0.81	1.26	0.93	0.74	1.16
Demographics																			
Age	1-year increase	1.10	1.08	1.11	1.09	1.08	1.11	0.97	0.96	0.99	0.96	0.94	0.97	1.08	1.06	1.09	1.07	1.06	1.09
Sex	Female/Male	2.41	2.06	2.81	2.40	2.03	2.84	1.38	1.19	1.60	1.32	1.12	1.56	1.29	1.12	1.50	1.16	0.99	1.35
Past or present history																			
Stroke	Yes/No	*			1.42	1.04	1.93	*			1.09	0.80	1.50	*			*		
AMI	Yes/No	*			2.30	1.48	3.57	*						*					
Cancer	Yes/No	*			*			*			1.21	0.94	1.57	*			0.72	0.55	0.95
Chronic conditions																			
Hypertension	Yes/No	*			1.06	0.89	1.26	*			*			*			*		
Overweight	BMI ≥25 kg/m^2^/<25	*			2.01	1.68	2.41	*			*			*			*		
Sleep disturbance	PSQI ≥5.5/<5.5	*			1.24	1.06	1.46	*			2.23	1.91	2.62	*			1.51	1.29	1.76
Poor vision	Present/Absent	*			*			*						*			1.43	0.91	2.25
Poor hearing	Present/Absent	*			1.40	1.09	1.79	*			1.13	0.87	1.47	*			*		
Geriatric syndromes																			
Depressive symptoms	GDS ≥6/<6	*			1.97	1.61	2.42	*			3.42	2.82	4.15	*			1.94	1.59	2.37
Cognitive impairment	MMSE ≤23/>23	*			*			*			1.24	0.88	1.74	*			1.83	1.35	2.49
Urinary incontinence	Present/Absent	*			1.37	1.14	1.64	*			1.29	1.07	1.55	*			1.36	1.13	1.63
Falls	Present/Absent	*			1.50	1.25	1.80	*			1.29	1.07	1.56	*			1.46	1.22	1.75
Psychosocial factors																			
Support by family members	Absent/Present	*			*			*			1.46	1.23	1.74	*			1.11	0.93	1.33
Support by friends	Absent/Present	*			1.28	1.06	1.53	*			1.39	1.16	1.67	*			1.05	0.87	1.25
Education	≤12 years/>12 years	*			*			*			*			*			1.16	0.98	1.38

ORs: odds ratios, CIs: confidence intervals, PCS: physical component summary, MCS: mental component summary, RCS: role component summary.

Asterisks show variables that were not included at *P* < 0.1 in sex- and age-adjusted logistic regression analysis for each component summary.

AMI: acute myocardial infarction, BMI: body mass index, PSQI: Pittsburgh sleep quality index, GDS: geriatric depression scale, MMSE: mini-mental state examination.

When the diabetes group was divided into those with diagnosed diabetes (425 individuals) and those with undiagnosed diabetes (147 individuals), the former subgroup, as compared with the referent group, had a significantly higher OR (1.49; 95% CI 1.16–1.91) only for low PCS, after adjusting for the same confounders listed in Table 2. Participants with undiagnosed diabetes had a similarly high, but statistically insignificant, increase in the OR (1.44; 95% CI 0.97–2.15, *P* = 0.072) for low PCS.

Table 3 shows potential confounding variables associated with low PCS in the 425 participants with diagnosed diabetes. After adjustment for independent variables significantly associated with low PCS in sex- and age-adjusted logistic regression analysis at *P* < 0.1, there was no significant increase in ORs for low PCS in any of the 3 models (ie, glycemic control [model 1], treatment regimen [model 2], and duration of diabetes [model 3]). Because people with an HbA1c less than 7.0% were more likely than those with an HbA1c of 7.0% or higher to receive lifestyle modification treatment (28.5% vs 8.6%, *P* < 0.001), we examined the interaction between HbA1c level and treatment regimen for low PCS. However, no interaction was identified (*P* = 0.351). In addition, no interaction was noted between HbA1c level and duration of diabetes (*P* = 0.683). Notably, with regard to AMI, overweight status, and falls, all models showed significant increases in OR with low PCS.

**Table 3.** ORs and 95% CIs for low PCS in 425 participants with diagnosed diabetes, in 3 models

Independent variables		Model 1			Model 2			Model 3		
		
		OR	95% CI		OR	95% CI		OR	95% CI	
Glycemic control	HbA1c <7.0% (274)	1								
	HbA1c ≥7.0% (151)	0.94	0.58	1.52						
Treatment regimen	Lifestyle modification (91)				1					
	Oral medication (286)				1.35	0.75	2.43			
	Insulin therapy (48)				1.48	0.64	3.43			
Diabetes duration	<10 years (151)							1		
	10–19 years (126)							0.89	0.51	1.58
	≥20 years (73)							0.71	0.36	1.39

Age	1-year increase	1.11	1.06	1.16	1.11	1.06	1.16	1.13	1.08	1.18
Sex	Female/Male	4.16	2.58	6.72	4.13	2.55	6.70	3.88	2.30	6.55
AMI	Yes/No	3.06	1.30	7.19	3.06	1.30	7.17	2.80	1.02	7.67
Overweight	BMI ≥25 kg/m^2^/<25	2.16	1.32	3.56	2.17	1.32	3.58	1.90	1.10	3.28
Cognitive impairment	MMSE ≤23/>23	1.62	0.70	3.76	1.61	0.70	3.74	2.07	0.83	5.14
Falls	Present/Absent	1.75	1.05	2.93	1.75	1.05	2.93	1.55	0.90	2.71

Figures in parentheses are numbers of participants.

ORs: odds ratios, CIs: confidence intervals, PCS: physical component summary.

Independent variables significantly associated with low PCS in sex- and age-adjusted logistic regression analysis at *P* < 0.1 are listed.

AMI: acute myocardial infarction, BMI: body mass index, MMSE: mini-mental state examination.

## DISCUSSION

This study has 3 main findings. First, the physical component of QOL, as evaluated by the SF-36, showed significantly more impairment among older adults with diabetes than among those without diabetes, even after adjusting for potential confounders. Second, people with diagnosed diabetes and those with undiagnosed diabetes were equally affected by low PCS; however, no impact was observed in any of the 3 SF-36 component summaries in the high HbA1c group. Third, there were no significant associations between HRQOL and disease-specific factors, including glycemic control, treatment regimen, and duration of diabetes.

We are the first to report the use of the 3-component model for HRQOL assessment of older adults. Median PCS score decreased with age, but median MCS score increased (Figure), as was reported in the general Japanese population.^20^ As for RCS, a decrease in the median was observed with increasing age in the present study. Although no comparable results have been reported, our result is understandable given that RCS represents roles in daily life as well as social functioning, which decrease with age in the general Japanese population.^20^

The diabetes group had a significantly higher prevalence of past or present history of stroke or AMI as compared with the non-diabetes group (Table 1). These chronic diseases can substantially decrease HRQOL.^10^^,^^12^ The diabetes group also had a significantly higher prevalence of geriatric syndromes, including depressive symptoms and urinary incontinence. Geriatric syndromes decrease HRQOL^13^ and are more likely to appear in elderly people with diabetes, due to diabetic macro- and micro-vascular complications, hyperglycemia, and hypoglycemia.^4^^,^^5^ Therefore, to assess the impact of diabetes on HRQOL in older adults, we considered these factors as potential multidimensional confounders, in addition to sex and age, in conjunction with psychosocial factors.

Our analysis using the 3-component model of SF-36 with multidimensional confounders revealed that the adjusted OR (1.48; 95% CI 1.18–1.84) for low PCS was significantly increased only in the diabetes group (Table 2). This result agrees with previous findings, which showed that older adults with diabetes had a higher risk of developing physical disability.^30^^,^^31^ Lower PCS can be interpreted as subjective difficulty with activities in everyday life (eg, walking, climbing, bathing), limitations in carrying out work or daily activities, or pain that interferes with work or daily activities. One pathway to low PCS would be the presence of cardiovascular disease and obesity. Both are associated with limited cardiopulmonary reserve and exercise tolerance and contribute to functional disability.^32^ In the present study, however, the adjusted OR for low PCS remained significantly higher than unity in the diabetes group, even after including AMI and overweight in multiple logistic regression analysis. Because of the association between diabetes and greater decline in leg muscle mass and strength,^33^ we surmise that diabetic neuropathy and microangiopathy is another pathway to low PCS. In the present study, we lacked data to confirm the association with neuropathy and microangiopathy, so further studies are needed to clarify this point. One study found that physical activity could help prevent functional limitations in older diabetic individuals with an average HbA1c of 8.5%.^31^ Although the average HbA1c among our diabetes patients was much lower, 6.8%, physical activity could effectively maintain physical function.

As compared with the referent group we found no decreases in MCS or RCS among the diabetes and high HbA1c groups. Our participants were independent people living in the community. None had undergone hemodialysis, only a small percentage had difficulties with vision, and less than 10% required insulin therapy. This suggests that the present participants were not severely impaired to the extent that mental health or social functioning was affected.

Of the 2 diabetes subgroups, persons with diagnosed diabetes had significantly higher prevalences of stroke, AMI, and low eGFR than did those with undiagnosed diabetes. However, after adjusting for these confounders the ORs for low PCS were very similar (1.49, 95% CI 1.16–1.91 vs 1.44, 0.97–2.15). This accords with the fact that median HbA1c did not differ between the subgroups (6.7% vs 6.8%). Because high HbA1c reflects chronic hyperglycemia, the undiagnosed diabetes group is at risk of developing diabetes and cardiovascular disease^18^; hence, proper management is recommended. We also evaluated HRQOL in the high HbA1c group (5.7 ≤ HbA1c < 6.5%). No significant correlation was found in any of the 3 SF-36 component summaries, as compared with the referent group (HbA1c <5.7%).

On the basis of differences in findings between the diabetes and high HbA1c groups, efforts to prevent progression to diabetes might be important for maintaining HRQOL in the elderly.

Glycemic control is an important indicator of diabetes treatment.^3^^–^^5^ In the present study HbA1c level (≥7.0% vs <7.0%) was not associated with a significant increase in OR for low PCS in the diagnosed diabetes group in multiple logistic regression analysis (Table 3). In addition, no significant interactions were found between HbA1c level and either treatment regimen or diabetes duration. As was the case for this study, some earlier reports^34^^,^^35^ found no significant effect of glycemic control on HRQOL, as evaluated by generic non-specific QOL questionnaires. However, all scores for the Diabetes Quality Of Life (DQOL) scale were substantially affected by HbA1c level,^36^ and the Elderly Diabetes Burden Scale (EDBS) revealed that higher HbA1c levels and hypoglycemia increased burdens such as dietary restrictions among diabetes patients.^37^ Thus, disease-specific QOL measurements might better detect differences related to glycemic control.

We found that treatment regimen and diabetes duration were not significantly associated with ORs for low PCS (Table 3). Previous reports were inconclusive: some reported that intensive therapies had no impact on QOL of diabetes patients,^38^ while others found that insulin use had a weak influence on HRQOL.^13^ Treatments more intensive than lifestyle modification, such as oral medication and insulin therapy, were associated with decreased HRQOL.^35^^,^^37^ In addition, previous findings on the association between duration of diabetes and HRQOL were inconsistent.^12^^,^^39^ These discrepancies might be due to differences in the age groups targeted, the treatment regimens compared, the questionnaires used for assessing HRQOL, treatment duration, and/or the different confounders considered simultaneously.

There are several limitations in this study. First, determination of diseases was based on a self-reported questionnaire and thus disease identification might not have been accurate. Specific tests for retinopathy and neuropathy were not performed. Second, we used a generic measurement to compare people with and without diabetes: disease-specific HRQOL instruments such as the DQOL^36^ and EDBS^37^ may provide more-sensitive information to assess troublesome symptoms and experiences related to diabetes and its treatment. Third, the study design was cross-sectional, which did not allow for determination of a causal relationship between diabetes and low HRQOL. Despite these limitations, this study has strengths in that obtained results were based on a large community-based population. Moreover, we were able to adjust for most of the factors associated with HRQOL that are especially important in elderly people.

In conclusion, older adults with diabetes are at high risk for poor physical HRQOL independent of demographic variables, history of disease, chronic conditions, geriatric syndromes, and psychosocial factors. To prolong healthy life expectancy, it is important to maintain physical functioning, in addition to promoting interventions aimed at reducing diabetic complications and geriatric syndromes. HbA1c level, treatment regimen, and diabetes duration were not related to low PCS, suggesting that diabetes prevention itself might have an essential role in maintaining HRQOL among the elderly.

## ONLINE ONLY MATERIALS

Abstract in Japanese.
