# Supplementary material for: Health-related Quality of Life (HRQOL) Decreases Independently of Chronic Conditions and Geriatric Syndromes in Older Adults With Diabetes: The Fujiwara-kyo Study
Source: J Epidemiol. 2014 Jul 5;24(4):259–66. doi: 10.2188/jea.JE20130131 (PMC4074629; doi:10.2188/jea.JE20130131)
Supplement: Abstract in Japanese. [file je-24-259-s001.pdf]

タイトル

糖尿病罹患高齢者の健康関連 QOL (HRQOL) は合併する慢性疾患や老年症候群と独立して低下している：藤原京スタディ

著者

根津　智子1、岡本　希1、森川　将行[2]、佐伯　圭吾[2]、大林　賢史[1]、冨岡　公子[1]、小松雅代[3]、岩本　淳子[4]、車谷　典男[1]

所属

[1] 奈良県立医科大学地域健康医学教室

[2] 堺市こころの健康センター

[3] 奈良県立医科大学看護学科

[4] 天理医療大学看護学科

本文

背景：高齢者の糖尿病が慢性疾患や老年症候群と独立して健康関連 QOL（HRQOL）低下に関連しているか否かを検討した研究は極めて乏しい。

方法：65 歳以上の高齢者 3,946 人を対象に、自記式質問紙と面接調査で糖尿病と慢性疾患および老年症候群に関する病歴を得るとともに、HbA1c と血糖値の血液検査を実施した。HRQOL は SF-36（包括的健康関連 QOL 評価尺度）を用いて評価し、低 HRQOL に関する調整オッズ比と 95%信頼区間を多重ロジスティック回帰分析で算出した。

結果：全対象者のうち 3,521 人は医師から糖尿病と診断されたことはなく、そのうちの HbA1c<5.7%であった 2,345 人を基準群とした。HbA1c ≥5.7% かつ HbA1c <6.5% であった 1,029 人の基準群に対する低 HRQOL の調整オッズ比を慢性疾患、老年症候群やその他の交絡要因を調整して求めたところ、SF-36 の 3 種類の component summary である PCS（身体的側面の健康観）、MCS（精神的側面の健康観）、RCS（役割/社会的側面の健康観）のいずれも有意な上昇はみられなかった。しかし、医師に糖尿病と診断されているかまたは HbA1c ≥6.5%であった 572 人の糖尿病群では、PCS に関する調整オッズ比は 1.48 (95% CI 1.18-1.84)と有意に高値であった。一方、糖尿病と診断され治療をしていた 425 人では、血糖コントロール、治療方法、罹病期間の違いにより PCS、MCS、RCS で低 QOL に関する有意なオッズ比の上昇はみられなかった。

結論：糖尿病罹患高齢者の身体的 QOL は慢性疾患や老年症候群と独立して低下していた。

キーワード：糖尿病、高齢者、老年症候群、慢性疾患、SF-36
